# Mini-laparoscopic hysterectomy for adenocarcinoma in situ of the uterine cervix using interchangeable 5-mm end effectors: a way to cross the line of minimally invasive surgery in gynaecologic oncology

**Published:** 2017-09

**Authors:** A Galvao, D Goncalves, Morgado Alexandre, H Ferreira

**Affiliations:** Centro Materno Infantil do Norte (CMIN) – Centro Hospitalar do Porto (CHP), Porto, Portugal, Resident of Gynaecology and Obstetrics CMIN |CHP; Centro Materno Infantil do Norte (CMIN) – Centro Hospitalar do Porto (CHP), Porto, Portugal, Director of Gynaecology Department CMIN |CHP; Life and Health Sciences Research Institute, School of Health Sciences, University of Minho, Braga, Portugal; ICVS/3B’s Associate Laboratory, Braga/Guimarães, Portugal; Department of Obstetrics and Gynaecology, Centro Materno Infantil do Norte, Centro Hospitalar do Porto, Porto, Portugal.

**Keywords:** Mini-laparoscopy, Laparoscopy, Hysterectomy, Cervical Cancer

## Abstract

The incidence of adenocarcinoma of the uterine cervix is increasing. It poses the affected women in risk and the definitive treatment requires hysterectomy. Here we describe a case of adenocarcinoma in situ of the uterine cervix successfully managed by minilaparoscopic hysterectomy using interchangeable 5-mm end effectors.

## Introduction

Adenocarcinoma *in situ* of the uterine cervix is a precursor of cervical adenocarcinoma and may coexist with both adenocarcinoma and high- grade squamous dysplasia. Its incidence has been increasing, even in developed countries (Liu et al., 2001; Sasieni et al., 2009). The difficulty in detecting precursor glandular lesions, the fact that they are mostly asymptomatic lesions and the low sensitivity of cytology are cause of great concern (Ruba et al., 2004). These lesions are often multifocal and there may be hidden disease in up to 60% of cases (Wright, 2003). The definitive treatment of this pathology is by extra fascial hysterectomy (ACOG Practice Bulletin Number 131, 2012). Over the years, many studies have tried to find more conservative approaches, especially for young women who have not yet fulfilled their reproductive needs, such as cervical conisation, but interrogations persist about carcinological safety (Soutter et al., 2001; Graesslin et al., 2006; Munro et al., 2017).

There is already plenty of literature on the comparison between laparotomy and laparoscopy in the approach of cervical cancer (Ramirez et al., 2006; Frumovitz et al., 2007; Malzoni et al., 2009; Pellegrino et al., 2009; Nam et al., 2012; Ghezzi et al., 2013). The benefits of laparoscopic surgery are undeniable, such as reduction of surgical trauma, less blood loss, precision in tissue manipulation, need for smaller incisions, less postoperative pain, faster patient recovery, shorter hospital stay and better aesthetic results in comparison to the conventional route (Koehler et al., 2012). As so, even radical hysterectomies are now performed by laparoscopy (Malzoni et al., 2009).

Nowadays, the technological development in this area has been such that it is already possible to use instruments of very small calibre, without disturbing the surgical technique (Ghezzi et al., 2011). Mini- laparoscopy consists of the use of trocars of 3 mm or lower calibre, with the only possible exception being the umbilical port (Ghezzi et al., 2005; 2008; 2009; Uccella et al., 2015; Setubal et al., 2017). Its advantages are related to the small incision sites, which result in a lower incidence of incision- related complications, such as hernia, infection or pain (Novitsky et al., 2005; Yamamoto et al., 2011). The operative time does not seem to be augmented because of the use of these low calibre instruments and it permits a refinement of the surgical movements (Fanfani et al., 2013). However, the use of 3 mm instruments has been limited by the required downsizing of the end effector, which compromises the strength that can be performed in procedures such as hysterectomy. To overcome this, instruments having a 2.9-mm shaft that are inserted percutaneously through the skin (like in a needle puncture) have been developed, having 5 mm effector tips that can be inserted by a trocar of 5 or more mm and be coupled at the end of the instruments: the 2.9 mm Percuvance^TM^ Percutaneous Surgical System (PSS) (The Percuvance^TM^ System, Teleflex Inc., USA) (Chang et al., 2016).

Our objective is to demonstrate the feasibility of the use of mini-laparoscopic hysterectomy interchangeable 5-mm end effectors in a case of adenocarcinoma *in situ* of the uterine cervix.

## Case report

A 53-year-old otherwise healthy post-menopausal woman with no previous surgeries and a body mass index of 21 kg/m^2^ was referred to our department for atypical squamous cells of undetermined significance in the Pap smear with a human papillomavirus test positive for the genotype 16. The patient had no symptoms. She underwent colposcopy with biopsy and the result was cervical intraepithelial neoplasia type 1 with suspicion of a glandular lesion. Cervical conisation was made using LASER and the histology revealed adenocarcinoma *in situ* of the uterine cervix. Her transvaginal ultrasound was normal.

After discussion of the therapeutic options with the patient, it was decided to perform a total hysterectomy with bilateral adnexectomy. Given the fact that she was a healthy woman with no previous abdominal surgeries, the approach chosen was mini- laparoscopy complemented with the Percuvance^TM^ system (The Percuvance^TM^ System, Teleflex Inc., USA) whose instruments are introduced through the abdominal wall without trocar.

**Figure 1:**
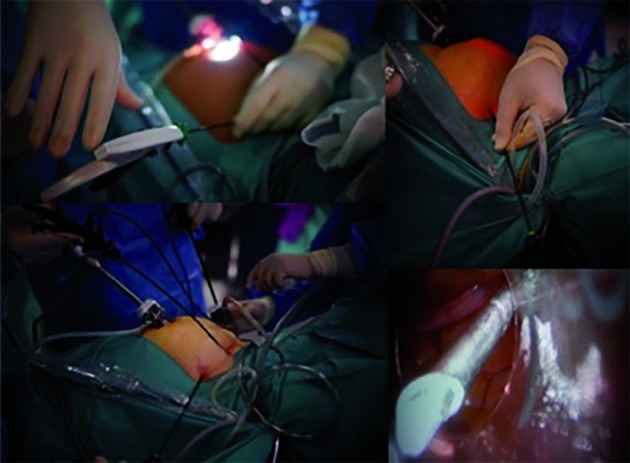
— Mini-laparoscopic hysterectomy complemented with the Percuvance^TM^ system (The Percuvance^TM^ System, Teleflex Inc., USA)

A mini-laparoscopic hysterectomy was performed. Under general anaesthesia, the patient was placed in the dorsal decubitus position with her arms alongside her body and her lower limbs in abduction. Pneumoperitoneum was achieved using a Veress needle placed in her umbilicus. One 6-mm trocar was placed at the umbilicus for the zero-degree laparoscope; two shafts of 2.9 mm Percuvance^TM^ Percutaneous Surgical System (PSS) (The Percuvance^TM^ System, Teleflex Inc., USA) were then inserted under direct visualization, one at the right anterosuperior iliac spine and another at the left anterosuperior iliac spine (figure 1).

In the supra-pubic midline a 3.5 mm anciliary trocar was inserted (30114GZL; Karl Storz mini- laparoscopy trocar set) to allow the use of the 3.5 mm bipolar coagulator (Karl Storz Robi) and the mini-laparoscopic needle holder (Karl Storz mini- laparoscopy set). The entire pelvis was inspected for invasive disease, which was not found. The end effectors were then introduced through the umbilical port and coupled to the rest of the instrument, with the possibility of changing the tool tips to grasp, cut, and manipulate tissue, as needed.

The procedure was undertaken using the 10 steps methodology: coagulation and section of the round ligament bilaterally; opening of the anterior leaf of the broad ligament; fenestration of the posterior leaf of broad ligaments to displace the ureter laterally; coagulation and section of the infundibulopelvic ligaments; coagulation and section of the utero- ovarian ligaments bilaterally; vesical-uterine and posterior dissection; uterine vessels dissection, coagulation and section; opening of the vagina; uterus and adnexa extraction by vaginal route; vaginal closure with laparoscopic figure of eight sutures. In the uterine vascular pedicle, coagulation was made with a 3.5 mm bipolar grasper (Karl Storz Robi) and to close the vagina a mini-laparoscopic needle holder assisted by a PSS instrument.

The estimated blood loss was 30 mL and the operative time was 60 minutes. There were no intra or postoperative complications and the patient was discharged home 48 hours after surgery without pain. Post-operatively, she needed 1000 mg of oral paracetamol 6 hours after the end of the procedure. No need to repeat the analgesia afterwards. On histology, there was evidence of low-grade squamous intraepithelial lesion, but with no evidence of glandular lesions. Post-operatively, the patient had a rapid recovery and she has been kept on follow-up with vaginal cytology, which was normal (after 6 and 12 months). At 12 months, the human papillomavirus test was negative. One year later, no visible scars were observed.

## Discussion

Endoscopic surgery has undergone a major and rapid development, which has led to increasing complexity of the procedures undertaken by this approach. The advances in surgical techniques and instruments have made mini-laparoscopy a viable way to perform hysterectomies (Ghezzi et al., 2008; Uccella et al., 2015). Even in the oncological field, there is now plenty of literature describing its use for endometrial, ovarian and cervical cancers (Fagotti et al., 2012; Garrett and Boruta, 2012; Ghezzi et al., 2009; 2015; Uccella et al., 2015).

Although the benefit of moving from laparoscopy to mini-laparoscopy is by no means as marked as the benefit of going from laparotomy to laparoscopy, the truth is that there also appear to be no disadvantages with its application: surgical time does not seem to increase, the rate of postoperative complications has been shown to be lower and cosmetic results are better. It is far more difficult to discuss whether we should use mini-laparoscopy, single-site surgery or even natural orifice transluminal endoscopic surgery as the best approach to our patients. All these techniques can be safely performed by skilled laparoscopic surgeons (Escobar et al., 2010; Fanfani et al., 2012; Şendağ F et al., 2017). There are also descriptions of procedures undertaken by robots (Boggess et al., 2008). One of the main advantages of mini-laparoscopy is that it allows a better dexterity and a surgeon that works with laparoscopy will be able to do mini-laparoscopy with little additional training (Fanfani et al., 2012; Uccella et al., 2015). Another advantage includes the reduced operative trauma and incisional site manipulation that occurs with 3 mm instruments compared to larger instruments (no incisional hernias have been described using mini-laparoscopy) and the few space occupied by this thinner instruments, that allows a much better view from the surgical field (Carvalho et al., 2011; Yamamoto et al., 2011).

The limitations of natural orifice surgery or single incision surgery include reduced surgeon ergonomics and difficulty in instrument triangulation, which can compromise the ability to suture correctly, endangering patient safety. With the use of the innovative Percuvance^TM^ system there are no such limitations and it is possible to maintain the advantages of mini-laparoscopic surgery without losing the precision and strength necessary to perform surgical procedures of some complexity. This novel device permits surgeons to maintain a standard setting and it represents a significant advance in minimally invasive surgery (Chang et al., 2016; Rossito et al., 2016; Romano et al., 2017). As described by other authors, we found that the Percuvance^TM^ system was a good alternative to a 5-mm port, the change of the end effectors was technically simple and we did not experience any disengagement of instrument tips during this procedure. (Chang et al., 2016). The only handicap is the limited variety of end effectors currently available.

Surgeons need to adapt themselves to this rapidly- changing world, deciding which new technology or technique they will adopt. The goal will be to make technology bring health benefits. We strongly believe that this was what happened with our patient, a healthy and asymptomatic woman who was cured from an oncologic disease, practically without having visible marks of the surgery she was submitted to. Some people may understand these marks as a pure aesthetic problem, but we believe this helps achieve a complete physical and psychological well-being.

## Declaration of Interests

The authors report no declarations of interest and confirm that they have obtained the written permission of the patient whose case is being presented.

## Conclusion

Percutaneous mini-laparoscopic hysterectomy is technically feasible and is a viable alternative to surgical treatment of women with adenocarcinoma *in situ* of the uterine cervix. It is never enough to emphasize its advantages, especially the minimization of surgical trauma, the refinement of the surgical movements and the higher patient satisfaction.

We hope our case to encourage more surgeons to learn and apply mini-laparoscopy, percutaneous systems and other new endoscopic techniques in favour of the patients.
